# Impact of climate and land use/land cover changes on malaria incidence in the Ecuadorian Amazon

**DOI:** 10.1371/journal.pclm.0000315

**Published:** 2024-04-19

**Authors:** Andrea L. Araujo Navas, Mark M. Janko, Fátima L. Benítez, Manuel Narvaez, Luis E. Vasco, Prakrut Kansara, Benjamin Zaitchik, William K. Pan, Carlos F. Mena

**Affiliations:** 1 Universidad San Francisco de Quito USFQ, Institute of Geography, Quito, Pichincha, Ecuador; 2 Duke Global Health Institute, Duke University, Durham, North Carolina, United States of America; 3 Department of Earth and Environmental Sciences, Faculty of Bioscience Engineering, Katholiek Universiteit van Leuven, Leuven, Belgium; 4 Department of Earth and Planetary Sciences, Johns Hopkins University, Baltimore, Maryland, United States of America

## Abstract

Malaria transmission is influenced by climate and land use/land cover change (LULC). This study examines the impact of climate and LULC on malaria risk in the Ecuadorian Amazon. Weekly malaria surveillance data between 2008 and 2019 from Ecuador’s Ministry of Public Health were combined with hydrometeorological and LULC data. Cross-correlation analyses identified time lags. Bayesian spatiotemporal models estimated annual LULC rates of change (ARC) by census area and assessed the effects on *Plasmodium vivax* and *Plasmodium falciparum* incidence. ARC for the five land cover classes (forest, agriculture, urban, shrub vegetation, water) ranged from −1 to 4% with agriculture increasing across areas. Forest and shrub vegetation ARC were significantly associated with both *Plasmodium vivax* and *Plasmodium falciparum*. Temperature and terrestrial water content showed consistent negative relationships with both species. Precipitation had varying effects on *Plasmodium vivax* (null) and *Plasmodium falciparum* (increase) incidence. Shrubs and forest expansion, increased temperature, and terrestrial water content reduced malaria incidence, while increased precipitation had varying effects. Relationships between malaria, LULC, and climate are complex, influencing risk profiles. These findings aid decision-making and guide further research in the region.

## Introduction

Between 2008 and 2012, Ecuador reduced the annual number of malaria cases from six to near zero (0.2) cases per 1000 inhabitants [1] reaching a pre-elimination stage in 2013. This occurred thanks to the SNEM (acronym in Spanish for “Servicio Nacional de Control de Malaria”) [2], the malaria control project in the Andean frontiers, and the Ministry of Health [3, 4]. International financing was also provided by the Global Fund in 2013, allowing for the distribution of insecticide-treated bed nets, rapid diagnostic tests, artemisinin-based combination therapy, and improving community epidemiological surveillance and education [5–7]. Nevertheless, the pathway towards elimination was interrupted following the withdrawal of Global Fund financing, and the government’s decision to disintegrate the SNEM. Within three years, cases rebounded, reaching an incidence of 3 cases per 1000 inhabitants in 2018, disproportionately affecting indigenous communities near the Peruvian border [8, 9].

In Ecuador, malaria is endemic in areas below 1500m of altitude in the Amazon region (e.g. the lower Napo and Pastaza regions) [10], with two predominant vectors of transmission, the female Anopheles mosquitoes from *An*. *Oswaldoi* [11, 12] and *An*.*benarrochi* species are found in the provinces of Orellana and Morona Santiago [11, 13, 14]. In Ecuador two pathogens are prevalent, *Plasmodium vivax* (87%) and *Plasmodium falciparum* (13%) [15].

Malaria outbreaks generally appear in wet periods of the year, generally from March to July, with mean precipitation and temperature of 294 mm and 26.3°C, respectively [10]. Humidity is constant throughout the year [10]. Additionally, temperature might influence the daily survival of adult mosquitoes, their embryogenesis, and the duration of the egg development stage [16]. Precipitation also drives mosquitoes’ daily survival by altering their population density and providing an optimal aquatic environment for their development [17], while also facilitating human movement in the region when water levels in the river increase due to increases in precipitation [9]. Soil moisture has also been shown to drive optimal sites for vector reproduction [18].

In the case of land use and land cover in the Amazon, forest is known to regulate soil and atmospheric temperature [3, 9, 11], limiting the presence of mosquito larvae, which normally prefer brushes or grass [17] that grow in forest fringes after a deforestation process [19]. Other research shows that agricultural activities can increase malaria transmission, depending on the type of crop [16]. Urban areas are related to low malaria risk if they are far from recent deforestation [1], as they create conditions inhospitable to mosquito larvae and reduce human exposure [20]. Nevertheless, urban infrastructure triggers the opening of roads, water pits, reservoirs, and other infrastructure, which could favor optimal habitats for mosquito reproduction [3], and increase the connections between humans and vectors. Vittor et al., [21] found that water body size and proximity to populations were significant determinants for mosquitoes’ presence and development [1, 22].

Considering that malaria ecology in the Amazon region is influenced by climatic and LULC variations over time by, altering the balance and context in which parasites and mosquitoes transmit and develop the disease [23]. Most of the malaria research in South America has focused on the whole Amazon region, or specific areas in Peru or Brazil, leaving a big gap in knowledge about the situation in the Ecuadorian Amazon, which is needed to guide malaria control and elimination planning. Leveraging malaria, climatic, and land use/ land cover data, we aim to understand the effects of climate and annual rates of change of LULC on malaria incidence in the Ecuadorian Amazon between 2008 and 2019 by (i) identifying specific time lags of high correlation between climate and malaria incidence, (ii) quantifying land use /land cover ARC in the study area, and (iii) estimating the effect of each LULC type and climate on malaria incidence.

## Materials and methods

### Study area and malaria incidence data

The Ecuadorian Amazon spans eastern Ecuador with an extension of about 116,500 km^2^. Approximately 80% of the area is covered by forest and natural ecosystems, with a typical tropical humid climate. The study area receives an average of about 3,200 mm of rainfall per year and has an average annual temperature that ranges from 19°C to 26°C. This region is divided into six administrative provinces and has a total of 1796 census areas and a population of around 740000 inhabitants [24]. A census area is a geographic division that contains approximately 80 to 150 households [24].

**Fig 1.**
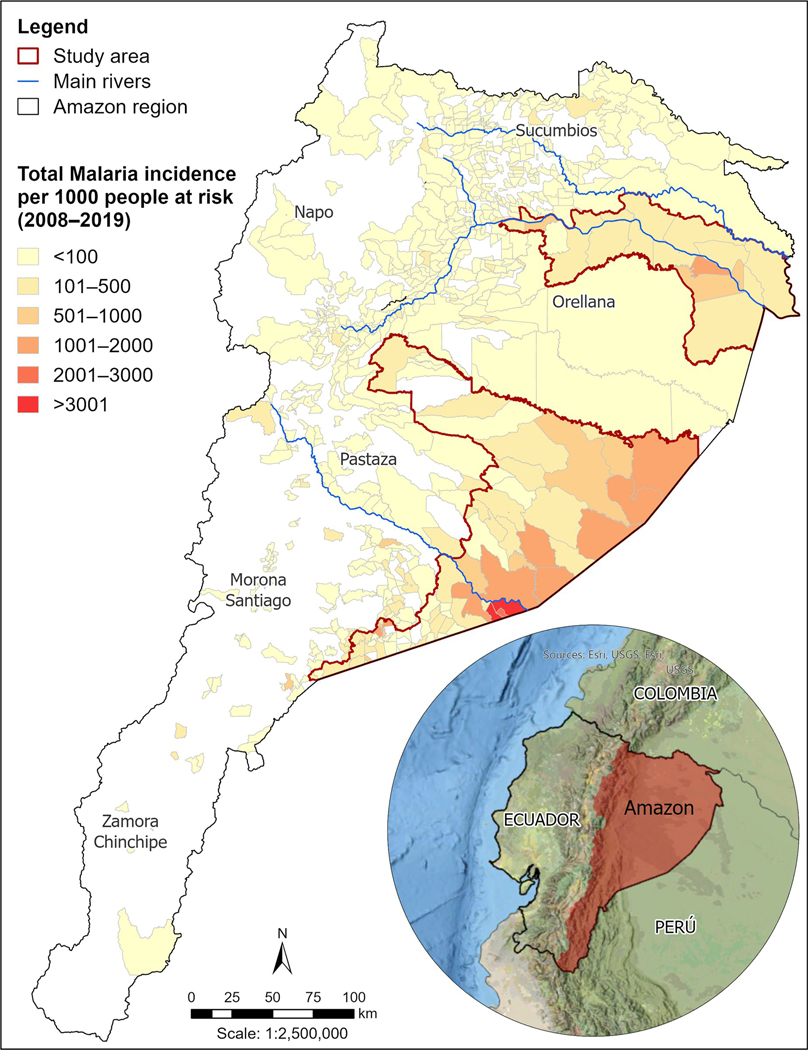
Spatial distribution of cumulative malaria incidence in the Ecuadorian Amazon region in the period 2008–2019. Main rivers were digitalized from open street maps. “Contains information from OpenStreetMap and OpenStreetMap Foundation, which is made available under the Open Database License”.

We obtained daily malaria case data from January 2008 to December 2019 from the Ecuadorian Ministry of Public Health. In this period, a total of 11,170 malaria cases, including 483 cases of *Plasmodium falciparum* (4%) and 10,687 cases of *P*.*vivax* (96%) were reported in the Ecuadorian Amazon region. Malaria cases were recorded at health centers whenever people with symptoms went there to be diagnosed. Malaria records include the location of the health center, test date, type of test, data of initial symptoms, *plasmodia* species, patient’s sex, age, and community locations (where the patient presumably contracted malaria). When a patient presented to a health post with symptoms of malaria (fever, shaking chills, sweating, headache, sickness, and dizziness), they were tested for malaria. Malaria diagnosis was primarily done using microscopy, although rapid diagnosis tests were also used in health centers where these were available [15]. Diagnosis methods were based on clinical and epidemiological criteria. Clinical criteria included fever, shaking chills, sweating, headache, sickness, and dizziness. Epidemiological criteria included exposure in the last 15 days to areas with high malaria transmission or people who suffered from malaria. Also, antecedents of blood transfusion and having received malaria medicine in the last four weeks were a criterion [15]. Cases were aggregated to the census area and epidemiological week levels. Fig 1 presents the census areas with the total number of malaria cases reported from 2008 to 2019 distributed in the whole Ecuadorian Amazon. The study area was selected based on census areas with cases higher than 5% of total malaria cases (11,170) during the period of analysis, but also included neighbor census areas with cases lower than 5% of total malaria cases, to maintain the diversity in our selection. We did this to have a data set including neighbor census areas with no cases. A total of 113 census areas are part of two main study regions, one at the north along the Napo River, and the other at the center and south of the Amazon in the Pastaza and Morona Santiago provinces.

One of the limitations in the study design is the fact that patients who reported malaria cases might not indicate the exact place where malaria was transmitted, but the place they think infection was the most probable. We assume this is the place where the person got infected. Another limitation is that the case detection is passive, meaning that asymptomatic infections were not considered.

### Environmental factors and descriptive variables

Factors capturing climate and LULC relationship with malaria incidence were defined using a combination of variables based on the literature. Hypotheses about these relationships are found in S1 Table. These hypotheses are part of a modeling exercise and are not designed to be tested as climate-environmental exposures on malaria. Whether or not covariates are expected to be associated with increased or decreased risk is shown in Table 1. The climate factor was defined using precipitation, temperature, soil water moisture, surface runoff, and total water storage derived from a Land Data Assimilation System (LDAS) developed for the Amazon region [9, 25]. Surface runoff is the aggregate quantity of water per unit of time moving through rivers or ravines. This measure encompasses the complete volume of water derived from various flows (including surface, subsurface, and baseflow) capable of reaching streams, thereby contributing to the overall outflows of the basins. We used the Noah-MP Land Surface model and HyMAP hydrological routing model [26] which together provide estimates of water fluxes distributed across the landscape and through the river network. The setup used for the meteorological and hydrological simulation comes from the previous study of Recalde-Coronel et al. [25] which evaluated the LDAS across Ecuador. The setup uses CHIRPS v.2. as the forcing precipitation dataset and GDAS for the rest of the meteorological variables, along with MODIS and SRTM for land use, vegetation status, and elevation.

LULC factor was defined using the percentage of shrub vegetation, agriculture, urban areas or settlements, and water bodies within a census area. LULC types were downloaded from the geoportal of the Ministry of Environment of Ecuador and were based on visual interpretation of ASTER and Landsat images for the years 2008, 2014, 2016, and 2018. These images provided a reasonable resolution of 30 meters and were freely available. For the year 2008, LULC was extracted from Landsat 7 ETM, whereas in 2013 the sensor used was Landsat 8 OLI. The first was performed radiometric corrections and the second no. The images were downloaded from http://glovis.usgs.gov/, http://earthexplorer.usgs.gov/ or https://espa.cr.usgs.gov. A summary of the used climatic and LULC covariates is presented in Table 1.

### Data preparation

The spatial and temporal units of analysis were the census area and epidemiological weeks, respectively. The shapefile from the census areas was obtained from the Statistical and Census National Institute (INEC, for its acronym in Spanish) for the year 2014 [27]. Our primary outcome variable is malaria incidence, calculated based on dividing the number of malaria cases stratified by species (*P*. *vivax* and *P*. *falciparum*) by the estimated census population for the years 2008–2019. Malaria cases and estimated census population are extracted for each census area and epidemiological week. Incidence is the number of new cases in a specified period (in our case we have one-year periods) over the population reported in the same period. The projected population was estimated by multiplying the population fraction and the population projections for each year at the *canton* level. The population fraction was calculated by dividing the population of each census area by the total population of the *canton* it belonged to. A *canton* is an administrative division bigger than the census area [24]; Yearly population projections at the *canton* level were calculated by the INEC, based on the 2010 census data [28]. We calculated the incidence rate for each census area and epidemiological week by dividing the number of cases and the projected population data.

Hydro-climate data was extracted for each census area by taking the mean of the climate variables within each census area and subsequently aggregated to epidemiological weeks. This was done for census areas that come at a higher scale than the hydro-climate spatial resolution. For census areas smaller than the hydro-climate variable resolution, the value for the census area centroid was extracted. The percentage of each LULC was calculated for each census area for the years 2008, 2014, 2016, and 2018.

### Cross-correlation analysis between malaria incidence and hydro-climatic variables

Cross-correlation analysis was conducted to understand the time-lagged relationship between weekly hydro-climate data and malaria incidence. A pre-whitening procedure was applied before cross-correlation calculations to avoid the risk of spurious cross-correlations [29–31]. Considering the *Anopheles* life cycle in aquatic habitats and the incubation period in humans, we worked with a maximum time lag of 12 weeks. Lags with the strongest correlation were selected for each hydro-climatic variable among the different census areas. Lagged hydro-climatic variables were related to the corresponding incidence rate. Cross-correlation results for *Plasmodium vivax* and *Plasmodium falciparum* are presented in Fig A and Fig B in S1 Text, respectively.

### LULC model

This model incorporates the global time effect of LULC among the different census areas. The linear model formulation for LULC is presented in Eq 1.


(1) $$LULC_{ik}=\alpha_{LULCC}+u_{i}+v_{i}k_{i}+\varepsilon_{ik}$$

$LULC_{ik}$ is the LULC percentage at the census area i and year k (2008, 2014, 2016, and 2018), $\alpha_{LULCC}$ is the fixed intercept. Here $u_{i}$ is the spatial random intercept that allows for the effect of time (measured by covariate k) to vary spatially. The spatial slopes from this model are then used as covariates in the malaria incidence model (Eq 3). The error term $\varepsilon_{ik}$ follows a normal distribution $N\left(0,\sigma_{\varepsilon }^{2}\right)$. Spatial dependence was modeled based on a conditional autoregressive structure (CAR) model [32].

### Malaria incidence model

Before modeling we first checked for collinearity. We found a high correlation between forest and agriculture (−0.8), terrestrial water content and soil moisture (0.68), and precipitation and surface runoff (0.65). We compared WAIC values, from two models, one including hydroclimatic variables, forest, and shrub vegetation, and the other including hydroclimatic variables and all LULC covariates, except forest. We did this as deforestation processes might be related to agricultural practices, urbanization, and water bodies change. We selected the model with the lowest WAIC. This model included precipitation, temperature, terrestrial water content, forest, and shrub vegetation. We then centered and scaled all the covariates by subtracting the mean and dividing it by the standard deviation.

We modeled malaria incidence rates using a Poisson regression model as follows:

(2) $$y_{it}∼Pois\left(\lambda_{it}\right)$$

Where the mean $\lambda_{it}$ is the expected malaria rate in census area i=1,…,m during epidemiological week t=1,…,T. We stratified by species of Plasmodia, *Plasmodium vivax*, and *Plasmodium falciparum*. We incorporated the data model into a log-linear regression, described below.


(3) $$log\left(\lambda_{it}\right)=\beta_{0}+u_{i}+u_{t}+x_{it}^{T}\beta +v_{it}^{T}\delta$$

Where $\beta_{0}$ corresponds to the fixed intercept, β and δ are fixed effects vectors capturing the effects of the hydroclimate ($x_{it}^{T}$) and the annual rates of change in LULC covariates ($v_{it}^{T}$) from the models described above. Finally, $u_{i}$ and $u_{t}$ are the random intercepts varying by space and time, respectively.

We used the Integrated nested Laplace approximation (INLA) for parameter estimation. We used the default parametrization given by INLA. The α intercept of the LULC model was assigned a Gaussian prior distribution with a mean of 0 and precision equal to 0.001. Minimally informative priors are specified on the log scale effect precision defined as $\tau =\frac{1}{\sigma^{2}}$. Thus, $\log\tau_{u}$, $\tau_{v}=logGamma(0.001,0.001)$. The precision for the unstructured spatial error $\varepsilon_{ik}$ was assigned a $\log\tau_{\varepsilon }=logGamma(1,0.0005)$. For the malaria incidence model, a non-informative normal distribution with a large variability N∼(0,0.0001) priors were specified on $\beta_{0}$, β *and* δ, while as in the LULC model, minimally informative priors were specified on the effect precision $\tau_{u}=\frac{1}{\sigma^{2}}$. Thus, $\log\tau_{u}=logGamma(0.001,0.001)$ for both, space and time.

### Ethical clearance

This study has been approved by the ethics committee from the San Francisco de Quito University (CEISH) review board, composed of Gulnara Patricia Borja Cabrera and Ximena Garzón Villalba, with the approval number 2021–156M and evaluation code IE03-EX232–2021-CEISH-USFQ. Consent to participate was not obtained as only cases from participants with symptoms were recorded in health centers. Moreover, we aggregated surveillance data at census area and epidemiological week level, which enabled full de-identification of individuals involved in the survey.

## Results

**Fig 2.**
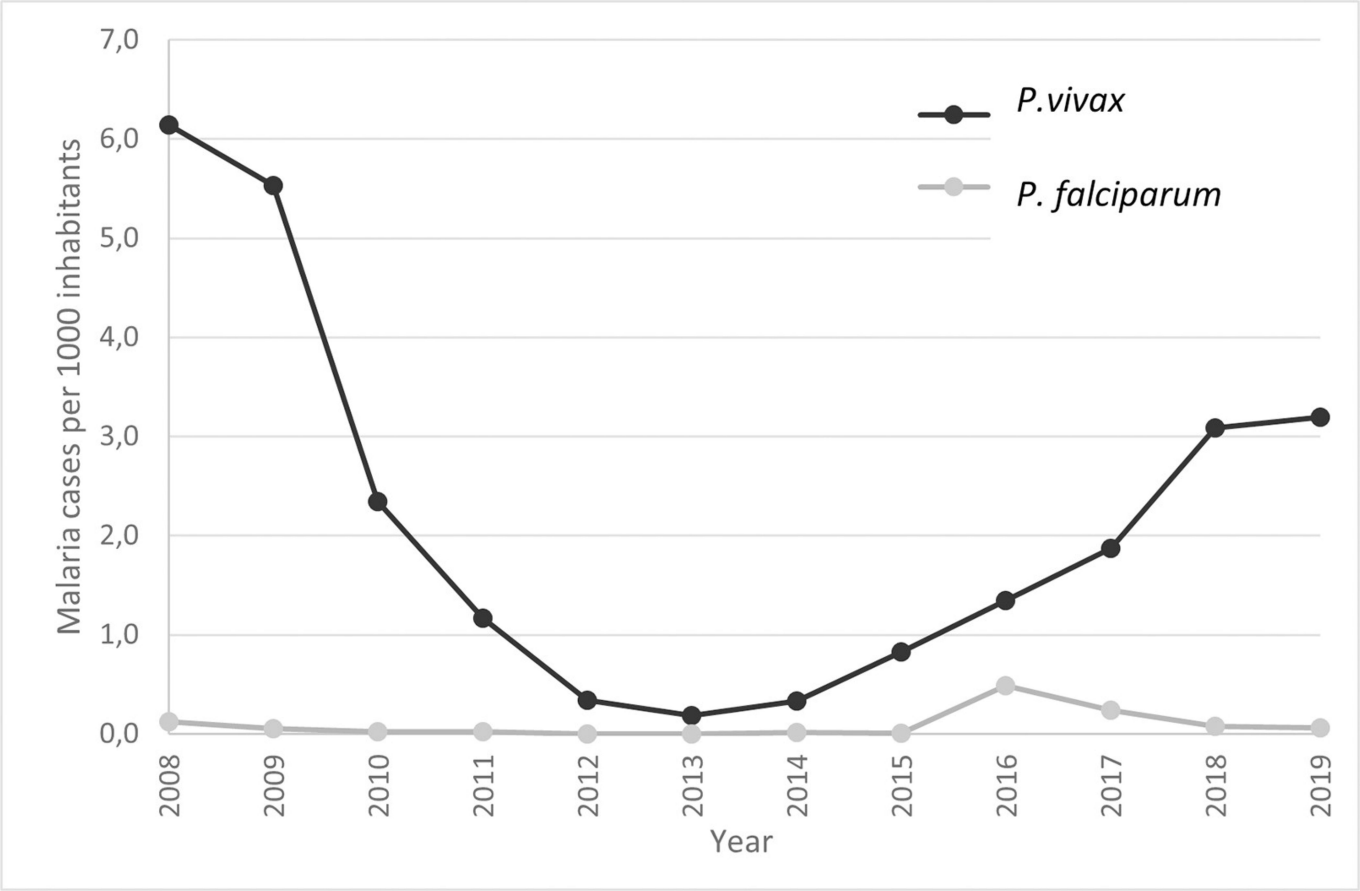
Number of malaria cases per 1000 inhabitants from 2008 to 2019.

As shown in Fig 2, this region experienced a decreasing infection period from around 6 cases per 1000 inhabitants in 2008, to almost zero cases per 1000 inhabitants in 2013. From 2012 to 2014, the number of cases per 1000 inhabitants was almost zero. Nevertheless, from 2015, cases per 1000 inhabitants increased from 1 to more than 3 in 2018 and 2019.

### Annual rates of LULC change

**Fig 3.**
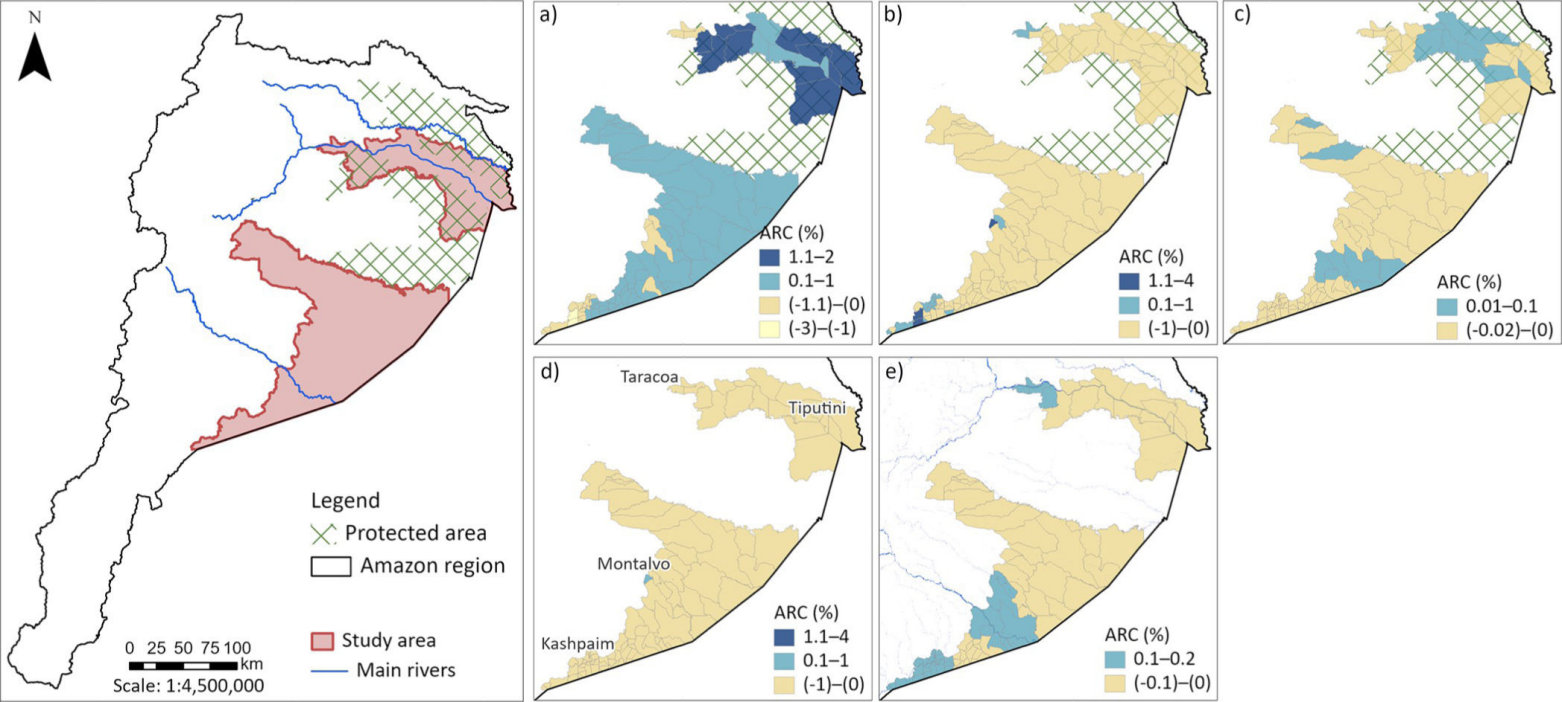
Spatial LULCC annual rates of change. a. Forest, b. Agriculture, c. Shrub vegetation, d. Urban Areas, e. Water Bodies. Main rivers were digitalized from open street maps. “Contains information from OpenStreetMap and OpenStreetMap Foundation, which is made available under the Open Database License”.

Forest cover expansion was observed in the north part of the study area, with rates of change varying from 1–2% per year. Some of these areas correspond to protected areas. Conversely, in the south, we observed annual rates of forest cover loss ranging from 1–3%, where agriculture and urbanization are expanding (Fig 3A) [33]. For example, agricultural land cover is expanding at rates between 1 to 4% per year in census areas concentrated in the south, particularly in the town of San Jose de Morona, which is close to the Peru border. Conversely, agricultural land cover is contracting at rates up to 1% per year in almost all the study areas (Fig 3B). Shrub vegetation is being lost at a rate of up to 0.02% per year in most of the study area, but is expanding at a rate of up to 0.1% in some central census areas (Fig 3C). Regarding urban areas, Taracoa (ARC), Tiputuni (ARC), Montalvo (ARC), and Kashpain (ARC) are expanding at rates from 1 to 4% per year. Urban areas are also contracting at rates of up to 1% in the rest of the study area (Fig 3D). Finally, a decrease and increase of water bodies was observed at rates up to 0.01% and 0.2%, respectively (Fig 3E).

### Influence of covariates on malaria incidence (fixed effects model)

#### Plasmodium vivax relationship with climate and LULC ARC.

Model results indicate that an increase of, 1.44°C in temperature, and 6.83 cm of terrestrial water content is associated with a decrease of 5.6% (Table 2: IRR 0.944; 95% UI 0.936–0.952), and 5.7% (Table 2: IRR 0.943; 95% UI 0.929–0.957) in *Plasmodium vivax* incidence, respectively. In the case of precipitation, its effect is null (Table 2: IRR 0.999; 95% UI 0.992–1.005). An increase of 1.3% ARC of forest and 0.02% of shrub vegetation, is associated with a decrease of 18.7% (Table 2: IRR 0.813; 95% UI 0.676–0.977) and 18.2% (Table 2: IRR 0.818; 95% UI 0.691–0.967), respectively, in *Plasmodium vivax* incidence. While an increase of 0.61% ARC in water bodies is related to an increase of 7.2% (IRR 1.072; 95% UI 0.897–1.282), in *Plasmodium vivax* incidence.

#### Plasmodium falciparum relationship with climate and LULC ARC.

Model results indicate that an increase of 3.7cm in precipitation shows an increase of 1.8% in *Plasmodium falciparum* incidence (Table 3: IRR 1.018; 95% UI 1.01–1.026). Conversely, an increase of 1.44°C in temperature and 6.83 cm of terrestrial water content, is associated with a 7.9% (Table 3: IRR 0.924; 95% UI 0.914–0.934) and 11.6% (Table 3: IRR 0.884; 95% UI 0.869–0.899) decrease in *Plasmodium falciparum* incidence, respectively. An increase of 1.3% ARC of forest and 0.02% of shrub vegetation ARC is associated with a decrease of 19.7% (IRR 0.803; 95% UI 0.664–0.969) and 18.4% (IRR 0.816; 95% UI 0.687–0.970), respectively in *Plasmodium falciparum* incidence. Finally, an increase of 0.61% ARC in water bodies is related to an increase of 6.3% (IRR 1.063; 95% UI 0.885–1.277), in *Plasmodium falciparum* incidence.

Graphs showing the effects of climatic and LULC variables on malaria incidence are shown as supplementary information (see S1 and S2 Figs).

## Discussion

Malaria is a forgotten disease in Ecuador that produces a big social and economic impact on indigenous communities and governments [10, 34]. Traditionally, malaria in Ecuador has received more attention in the coastal region of Ecuador [14, 35] and less in the Amazon [10], where the population density is considerably low. Studying patterns of malaria in Ecuador showed that deforestation and social vulnerability contribute to the increase of malaria incidence in the Amazon [36], especially in Amazon regions where the creation of roads, harvesting timber, developing farms and pasture, and oil extraction are predominant.

We show that in the Ecuadorian Amazon, climate has a strong role in the transmission of malaria in varying ways. In general, cross-correlation analyses showed low correlations between precipitation, temperature, and terrestrial water content with malaria incidence, especially for *Plasmodium falciparum*. This might be because of the relatively low incidence values along space and time. For instance, *Plasmodium vivax* and *Plasmodium falciparum* incidence values lower than 3%, are present in 61% and 83% of the census areas, respectively, and 95% and 83% of the epidemiological weeks, respectively.

Changes in LULC seem to be aligned with the Amazon situation. For instance, forest loss is located at the west border of the study region, where agricultural practices are common and increasing (Fig 3A and 3B). Conversely, gains in forest are present in protected and intangible areas such as Cuyabeno and part of Yasuni. Shrub vegetation is slightly growing in places where agricultural practices were already located (e.g. Nueva Loja, capital of Sucumbios province), but also at the south, in census areas along the river (Fig 3C). This might be because of the apparent increase/decrease of the river water level in this area (Fig 3E), exposing the natural vegetation.

Increases in temperature and terrestrial water content were associated with decreases in malaria incidence for both, *Plasmodium vivax* and *Plasmodium falciparum* [37]. As Zaitchik et al., [38] mentioned, the probability that mosquitoes will be found in a potential breeding site decreases with high temperatures. This is because too-warm conditions would bring disruption to some important life cycle phases of vector development. For instance, Stratman-Thomas and Warren in 1940 [39] showed that at all stages of *Plasmodium vivax* life cycle development, the parasite was more susceptible to unfavorable high temperatures (>37.5°C). Pathak et al. [40], also showed that increasing temperatures (>28°C) were detrimental to *Plasmodium falciparum* declines as well, due to the decrease in the mean proportion of mosquitoes infected with oocysts, and infectious with sporozoites. In the study area, temperature ranges from 16.26°C to 28.45°C, optimal for both parasites to survive. Nevertheless, when looking at the data, we found that both Plasmodia incidence rates reached a maximum of 163.9 (*Plasmodium vivax*) and 57.14 (*Plasmodium falciparum*) cases per 1000 inhabitants at temperatures higher than 25°C, while for temperatures from 20 to 25°C, *Plasmodium vivax* and *Plasmodium falciparum* incidence rates reached higher values of 345 and 633 cases per 1000 inhabitants, respectively. Temperature not only affects parasite sporology but also mosquito immunological or physiological changes [41]. Unfortunately, in the Ecuadorian Amazon, there is a large gap in understanding the biology and ecology of possible vectors, including *Anopheles oswaldoi* and *Anopheles benarrochi B*, which are the only vectors that have been encountered and studied in the study area [12, 13, 36]. Thus, although temperature is in an acceptable range for the *Anopheles oswaldoi* mosquitoes to survive [10], this relationship needs to be further studied.

In the case of terrestrial water content, too-wet conditions could limit the presence of mosquitoes in potential breeding sites [38, 42]. This was demonstrated by the study of Gunderson et al., [9], where findings showed that increases in soil moisture produced decreases in *Plasmodium vivax* and *Plasmodium falciparum*, *c*onsidering that terrestrial water content is the summation of soil moisture, root zone soil moisture, groundwater, vegetation stored water, river and water ponds water [43]. By observing our data, we see that lower incidences are present at higher TWC values. For instance, *Plasmodium vivax* and *Plasmodium falciparum* incidences reach values of 109.32 and 43.14 cases per 1000 inhabitants, respectively at TWC values higher than 5.8 m/week, while for lower values incidences are higher, 345 and 633 cases per 1000 inhabitants, respectively.

Precipitation determines available breeding habitats for mosquitoes [19] by feeding pools or standing water. We found different effects of precipitation on both plasmodia. Precipitation has a null effect on *Plasmodium vivax* and a minor increase in *Plasmodium falciparum* (1.8%) incidence. The null effect produced on *Plasmodium vivax* might be due to the homogenous rainfall regime (>3000 mm) in the Amazon plain throughout the year [44] (see S3 and S4 Figs). A higher influence of precipitation on *Plasmodium vivax* could probably occur in regions where water-limiting environments are more likely to be found [45]. Nevertheless, in places where precipitation is relatively constant throughout the year [46], like in the Ecuadorian Amazon plain, *Plasmodium vivax* dynamics might not be clear enough. A general negative effect of precipitation on *Plasmodium vivax* was found by Carrasco-Escobar et al., [41] for a time period series. This was attributed to high precipitation related to higher mortality of mosquito larvae. Nevertheless, as in this study, they found that the effect of precipitation was higher for *Plasmodium falciparum* than for *Plasmodium vivax*, attributed to the formation of optimal mosquito breeding sites for this parasite species vector, *Anopheles darlingi*, known to prefer rain puddles habitats [47]. In the case of *Plasmodium falciparum*, the effect of precipitation is positive, this was also shown in the study of Guarda et al., 1999, [37] where peaks in rainfall coincided with peaks in *Plasmodium falciparum* cases.

Regarding the effect of LULC, we found that an increase in malaria incidence was associated with forest decrease. Experiences in other Amazon countries show the same relationship [16, 20, 21, 48, 49]. For instance, Sawyer 1995 [49], stated that recent deforestation stages increase malaria prevalence due to the expulsion of wild animals from their habitats [48], which would provide alternative blood-meals for mosquitoes. Also, deforested places presented a higher *Anopheles darlingi* larval presence [21] and a biting rate 278 times higher than forested areas [16]. Agriculture and urbanization are also linked with deforestation processes, which are known to increase malaria risk [50, 51]. Fragmentation processes in the Ecuadorian Amazon could be caused by agriculture, especially in the northern Amazon region, produced by the increase of cattle raising, slash and mulch agricultural practices [46], and increasing large-scale commercial agriculture [52]. All of these could promote the creation of vector habitats [1, 16, 21] and mosquito breeding sites [53]. Also, recent urbanization has expanded the forest frontier, producing an increase in malaria rates [20].

Water bodies have a positive effect on both plasmodia, perhaps due to their increase when increasing deforestation and land clearing. Nevertheless, this effect is not significant, maybe due to the relatively minor annual rate of change ranging from 0.01 to 0.2%. The water bodies effect is related to the increase of vector-breeding sites while increasing the number of wetlands, fishponds, and shallow shady water, which seem to be present in many parts of our study area [1, 21]. Unlike the other LULC variables, shrub vegetation has a negative effect on both plasmodia. This is something that needs to be further explored, especially the characterization of different vegetation types, which have different origins (i.e. natural vegetation or regrowing vegetation after deforestation), structure, and shading, which might affect mosquito feeding, resting, and reproduction [54].

We identified four limitations, the first is related to the lack of data on interventions as environmental effects could be different when interventions are deployed [2]. The second is that we ignored socio-economic factors such as household conditions and demographic characteristics, which are also important factors to characterize malaria incidence. Leaving aside these variables could bias the results, leading to inaccurate interpretations about the significance and direction of the relationship among variables. The inclusion of these variables in the future could improve the precision of the estimates, and consequently, the understanding of the variable’s role in malaria incidence. Third, our results show a single effect value of each variable on malaria incidence, which could hide the real spatial and temporal effects, thus, a more detailed spatial-temporal model is needed to better understand malaria incidence dynamics throughout time and space. Finally, changes in land use and land cover through the years could not be captured due to the lack of information for each year. We think yearly changes might not be as detectable as long-period changes, thus, this would not be a strong limitation in the study. Besides, estimating the rates of change is our primary interest-measured variable.

This research informs decision-makers in different ways. Public health institutions such as the Ministry of Health and its sub-directions could be informed about how the climate and LULC affect malaria incidence in the study area. Although results inform in a general context the influence of LULC and climate, this might be the base for further studies, which could communicate and address efforts in the months or periods of the year when increases or decreases in incidences occur, and at which specific administrative areas. Not only the Ministry of Health could benefit from this research, but also leaders from indigenous associations, community leaders, health promotors, and other public health researchers. In particular, community leaders and health promotors could directly inform the community about forest and natural vegetation conservation strategies, needed to improve the community’s health and decrease malaria incidence.

## Conclusions

The results presented in this study provide a guide to understanding the overall dynamics of malaria incidence as a function of climate and LULC. In general, the effects of almost all variables were similar for both plasmodia, except precipitation. Different climate and LULCC effects occur due to the different habitat conditions needed by vectors and parasites. This suggests that by implementing climatic and LULCC strategies (e.g. surveillance systems) both plasmodia could be managed, and integrated into a public health program. This research informs healthcare providers, public health authorities and professionals, decision-makers, scientists, and the public in general, on the relevant factors that influence malaria incidence. This could be used to generate public health policies to understand and reduce the impacts of possible transmission events. This research also serves as a base for further studies analysing in detail the spatio-temporal dynamics of climate and LULCC on malaria incidence.

## Supplementary Material

Climate and LULC fixed effects for P.falciparum incidence model, posterior mean, and posterior 95% credible interval. **S1 Fig.** Climate and LULC fixed effects for *P*.*vivax* incidence model, posterior mean, and posterior 95% credible interval.

Climate and LULC fixed effects for P.vivax incidence model, posterior mean, and posterior 95% credible interval **S2 Fig.** Climate and LULC fixed effects for *P*.*falciparum* incidence model, posterior mean, and posterior 95% credible interval.

Cross-correlation analysis and results. **S1 Text.** Cross-correlation analysis and results.

Hypothesis about the relationship between hydro-climatic and LULC variables. **S1 Table.** Hypothesis about the relationship between hydro-climatic and LULC variables.

Climate spatial trends **S4 Fig. Climate spatial trends.** a) Average Precipitation, b) Average Temperature, c) Average Terrestrial Water Content.

Climate temporal trends. **S3 Fig. Climate temporal trends.** a) Average Precipitation, b) Average Temperature, c) Average Terrestrial Water Content.

## Figures and Tables

**Table 1.** Environmental and hydro-climatic variables.

	Variables	Indicator	Units	Spatial resolution	Temporal resolution	Source	Associated risk
Climate	Precipitation (P)	Accumulated Precipitation at centroids	mm	0.05°	weekly	CHIRPS-2.0/LDAS	Increase / Decrease
	Temperature (T)	Mean temperature at centroids	°C	0.1°	weekly	LDAS	Increase
	Soil water moisture (SWC)	Mean soil moisture	m^3^/m^3^ per week	0.1°	weekly	LDAS	Increase
	Total water storage (TWS)	Proxy for flooded areas (mean)	mm per week	0.1°	weekly	LDAS	Increase
	Surface runoff (QS)	Mean water runoff	mm per week	0.1°	weekly	LDAS	Increase / Decrease
Land Use and Land cover	Agriculture (A)	Percentage of agriculture	percentage	30 m	2008, 2014, 2016, 2018	Ministry of Environment	Increase
	Shrub vegetation (SV)	Percentage of bush	percentage	30 m	2008, 2014, 2016, 2018	Ministry of Environment	Increase
	Urban areas (UA)	Percentage of urban Areas or settlements	percentage	30 m	2008, 2014, 2016, 2018	Ministry of Environment	Increase / Decrease
	Water bodies (WB)	Percentage of water Bodies	percentage	30 m	2008, 2014, 2016, 2018	Ministry of Environment	Increase

**Table 2.** Climate and LULC fixed effects for Plasmodium vivax incidence model, posterior mean, and posterior 95% credible interval.

Parameters	IRR	2.5%	97.5%
Intercept	0.345	0.292	0.407
Precipitation*	0.999	0.992	1.005
Temperature*	0.944	0.936	0.952
Terrestrial Water Content*	0.943	0.929	0.957
Forest *	0.813	0.676	0.977
Shrub vegetation*	0.818	0.691	0.967
Water Bodies*	1.072	0.897	1.282
IRR: Incidence Rate Ratio			

* Standardized variables.

**Table 3.** Climate and LULC fixed effects for *P*.*falciparum* incidence model, posterior mean, and posterior 95% credible interval.

Parameters	IRR	2.5%	97.5%
Intercept	0.336	0.283	0.399
Precipitation*	1.018	1.010	1.026
Temperature*	0.924	0.914	0.934
Terrestrial Water Content*	0.884	0.869	0.899
Forest *	0.803	0.664	0.969
Shrub vegetation*	0.816	0.687	0.970
Water bodies*	1.063	0.885	1.277
IRR: Incidence Rate Ratio			

* Standardized variables.

## Data Availability

Data sets are available in the Open Science Framework repository (OSF) as part of the Ecuador malaria data project osf.io/svfp9.
