# Supplementary figures and images for: Impact of climate and land use/land cover changes on malaria incidence in the Ecuadorian Amazon
Source: PLOS Clim. Author manuscript; Available in PMC 2024 Jul 18. (PMC11257155; doi:10.1371/journal.pclm.0000315)

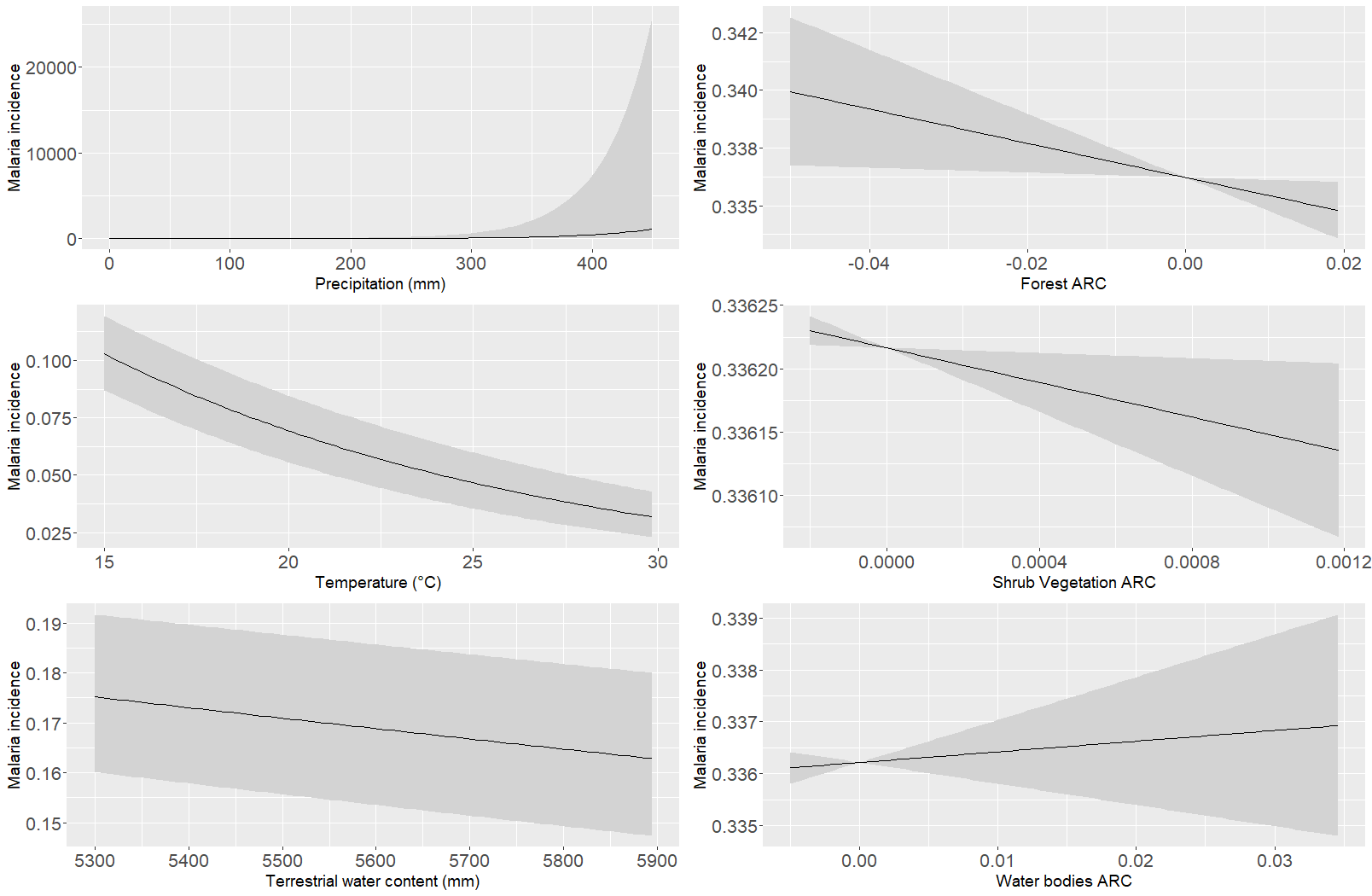

Supplement: Climate and LULC fixed effects for P.falciparum incidence model, posterior mean, and posterior 95% credible interval. — S1 Fig. Climate and LULC fixed effects for P.vivax incidence model, posterior mean, and posterior 95% credible interval. [file NIHMS2004284-supplement-Climate_and_LULC_fixed_effects_for_P_falciparum_incidence_model__posterior_mean__and_posterior_95__credible_interval_.tif]

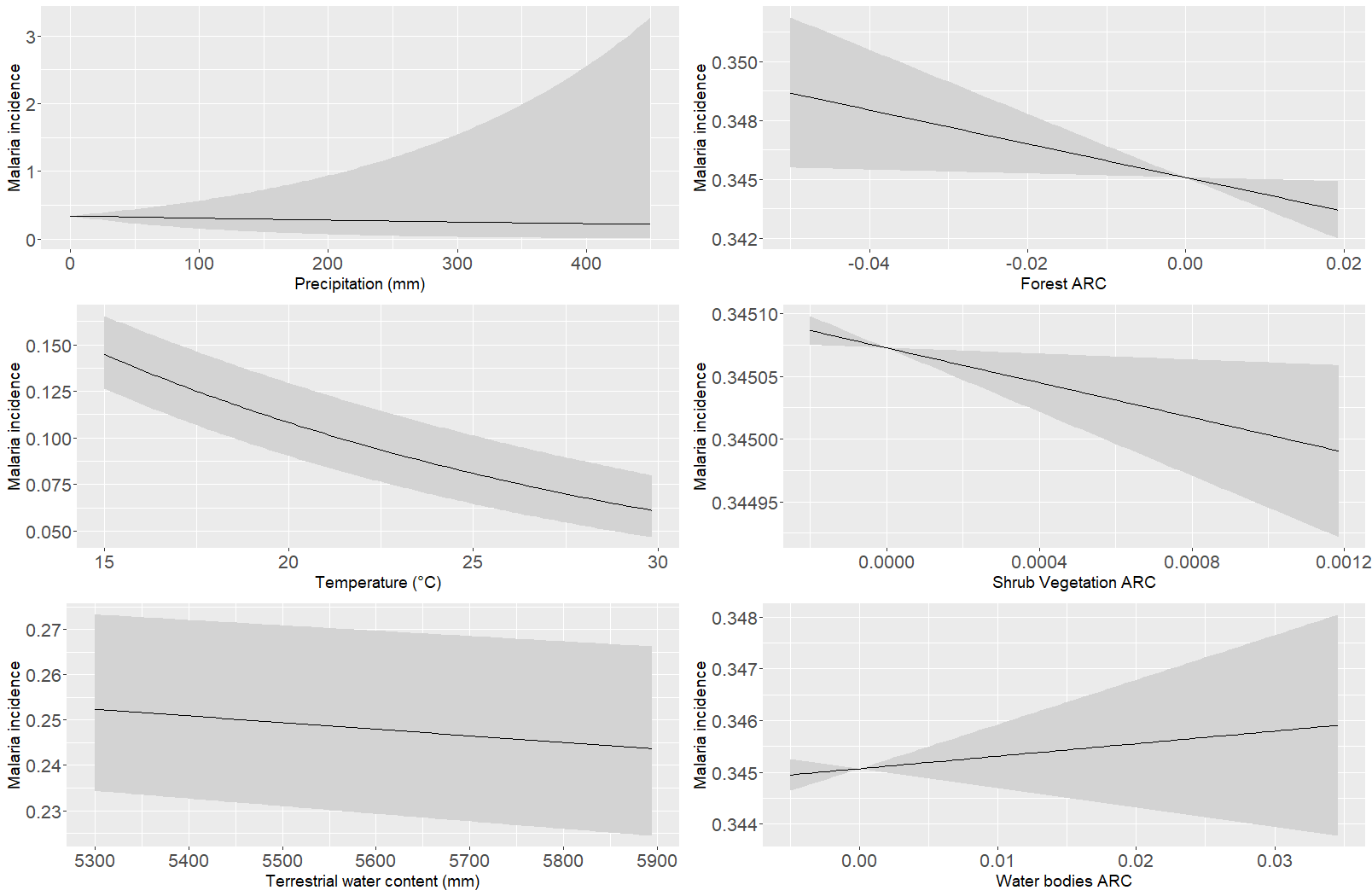

Supplement: Climate and LULC fixed effects for P.vivax incidence model, posterior mean, and posterior 95% credible interval — S2 Fig. Climate and LULC fixed effects for P.falciparum incidence model, posterior mean, and posterior 95% credible interval. [file NIHMS2004284-supplement-Climate_and_LULC_fixed_effects_for_P_vivax_incidence_model__posterior_mean__and_posterior_95__credible_interval.tif]

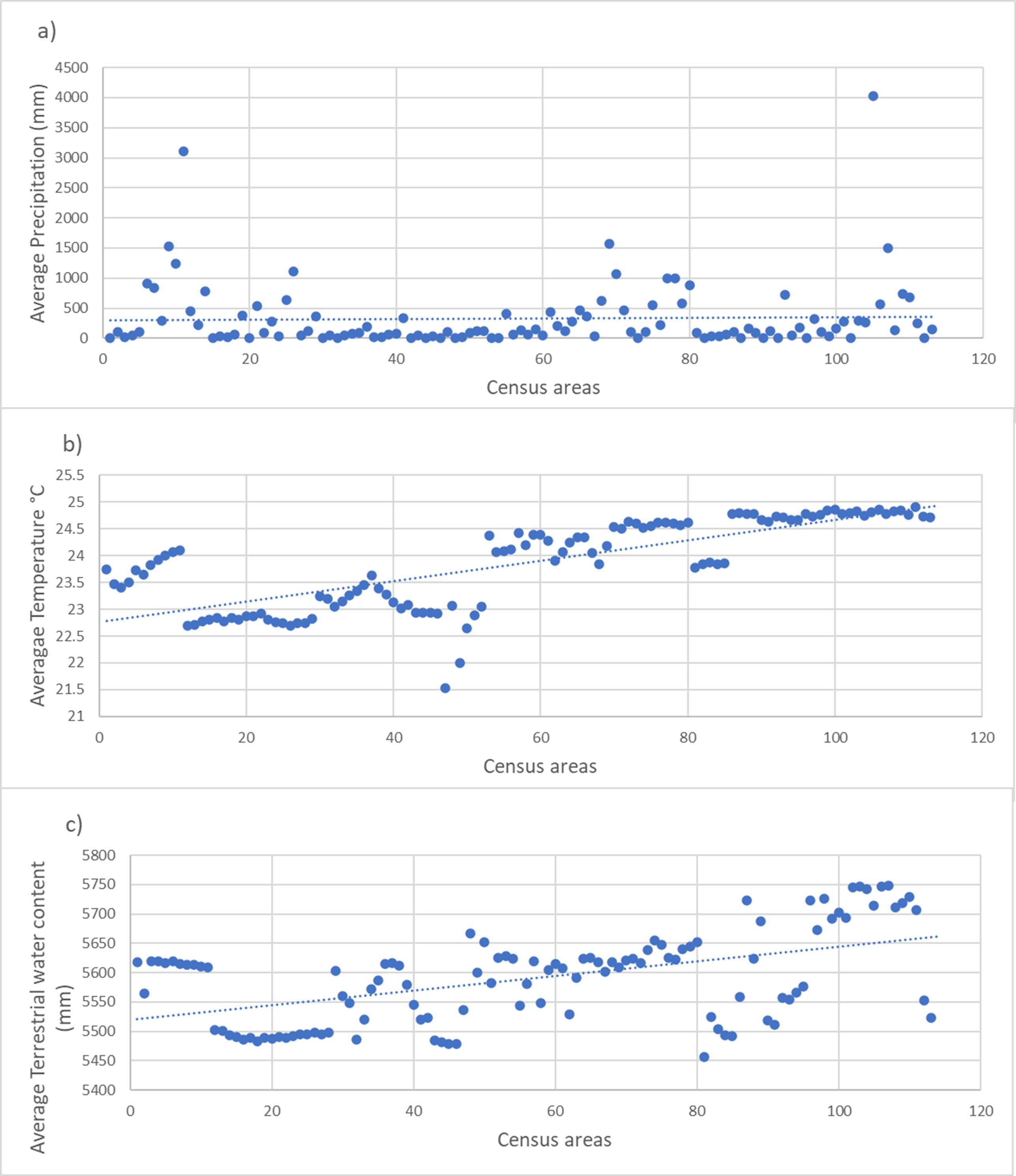

Supplement: Climate spatial trends — S4 Fig. Climate spatial trends. a) Average Precipitation, b) Average Temperature, c) Average Terrestrial Water Content. [file NIHMS2004284-supplement-Climate_spatial_trends.tif]

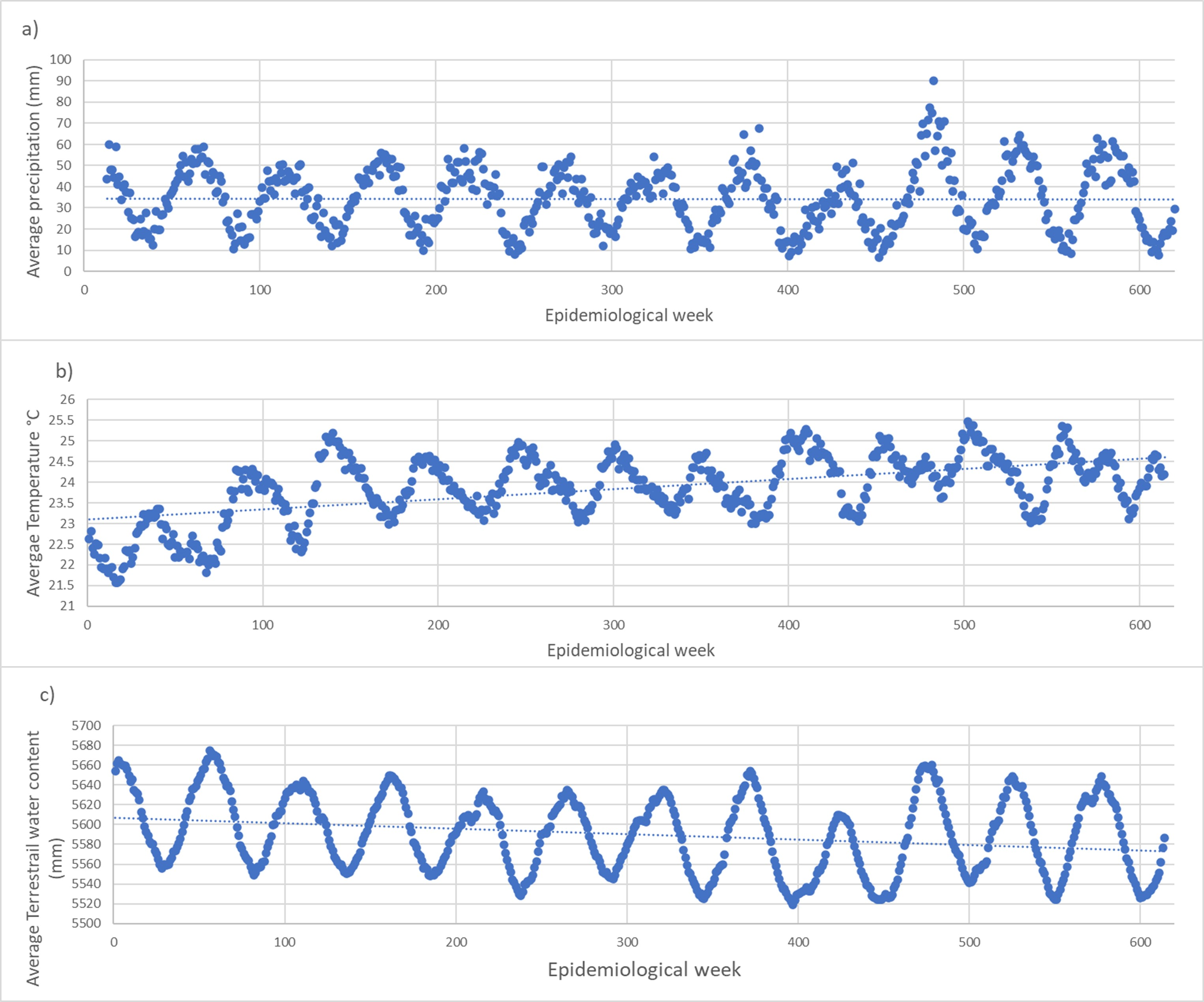

Supplement: Climate temporal trends. — S3 Fig. Climate temporal trends. a) Average Precipitation, b) Average Temperature, c) Average Terrestrial Water Content. [file NIHMS2004284-supplement-Climate_temporal_trends_.tif]
