# Supplementary material for: Impact of climate and land use/land cover changes on malaria incidence in the Ecuadorian Amazon
Source: PLOS Clim. Author manuscript; Available in PMC 2024 Jul 18. (PMC11257155; doi:10.1371/journal.pclm.0000315)
Supplement: Cross-correlation analysis and results. — S1 Text. Cross-correlation analysis and results. [file NIHMS2004284-supplement-Cross-correlation_analysis_and_results_.docx]

**Cross-correlation analysis**

These maps show the maximum correlation value for each census block and the lag time where this value occurs. In general, correlation values for both plasmodia are low. Still, in the case of Plasmodium vivax, most of the census areas have positive or negative correlation values ranging from -0.14 to 0.25. In contrast, for *Plasmodium falciparum* only the census areas near the frontier border have negative and positive correlation values from -0.25 to 0.2.

In general, we observe no correlation between *Plasmodium vivax* incidence and precipitation in most of the census areas (Figure 1a). A positive correlation with temperature and soil moisture is present in a few census areas in the north and south regions (Figure 1b and Figure 1c, respectively). Positive and negative correlations with terrestrial water content and runoff were found in a few heterogeneous census areas (Figure 1d, 1e).

In the case of *Plasmodium falciparum,* its correlation with precipitation and temperature is null for the majority of census areas (Figures 2a and 2b). In contrast, its correlation with soil moisture, terrestrial water content, and runoff is positive and negative in a few heterogeneously located census areas (Figure 2c, 2d, 2e).


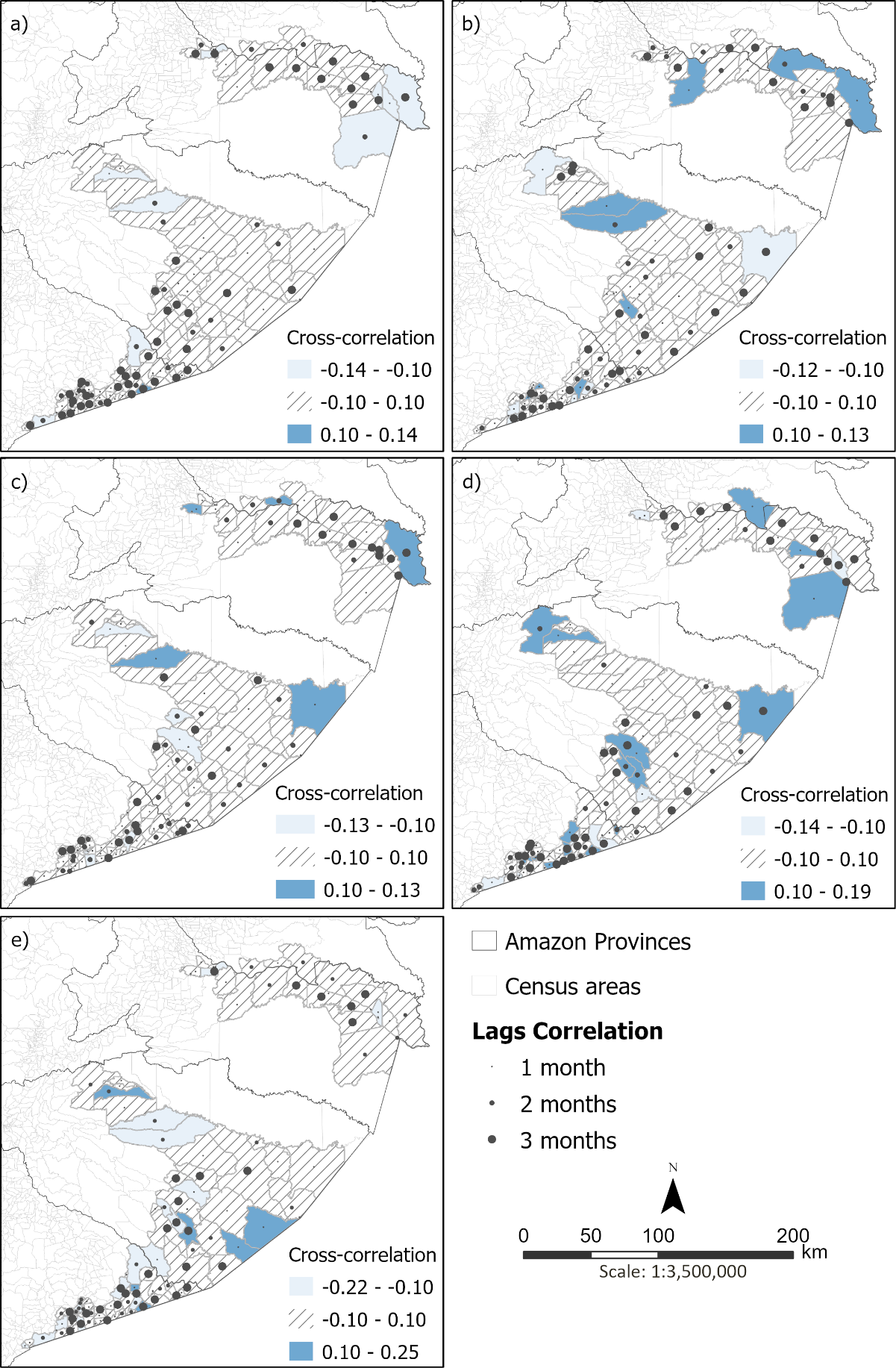


*Fig A. Cross-correlation values between Plasmodium vivax incidence and a) Precipitation, b) Temperature, c) Soil Moisture, d) Terrestrial Water Content, and e) Runoff.* **Provinces and census areas borders were obtained from the public domain source** [**https://www.ecuadorencifras.gob.ec/documentos/web-inec/Geografia_Estadistica/Micrositio_geoportal/index.html**](https://www.ecuadorencifras.gob.ec/documentos/web-inec/Geografia_Estadistica/Micrositio_geoportal/index.html)**.** **Terms of use are described here** [**https://www.ecuadorencifras.gob.ec/documentos/web-inec/Geografia_Estadistica/Micrositio_geoportal/marco-geoestadistico-2022.html**](https://www.ecuadorencifras.gob.ec/documentos/web-inec/Geografia_Estadistica/Micrositio_geoportal/marco-geoestadistico-2022.html)

*
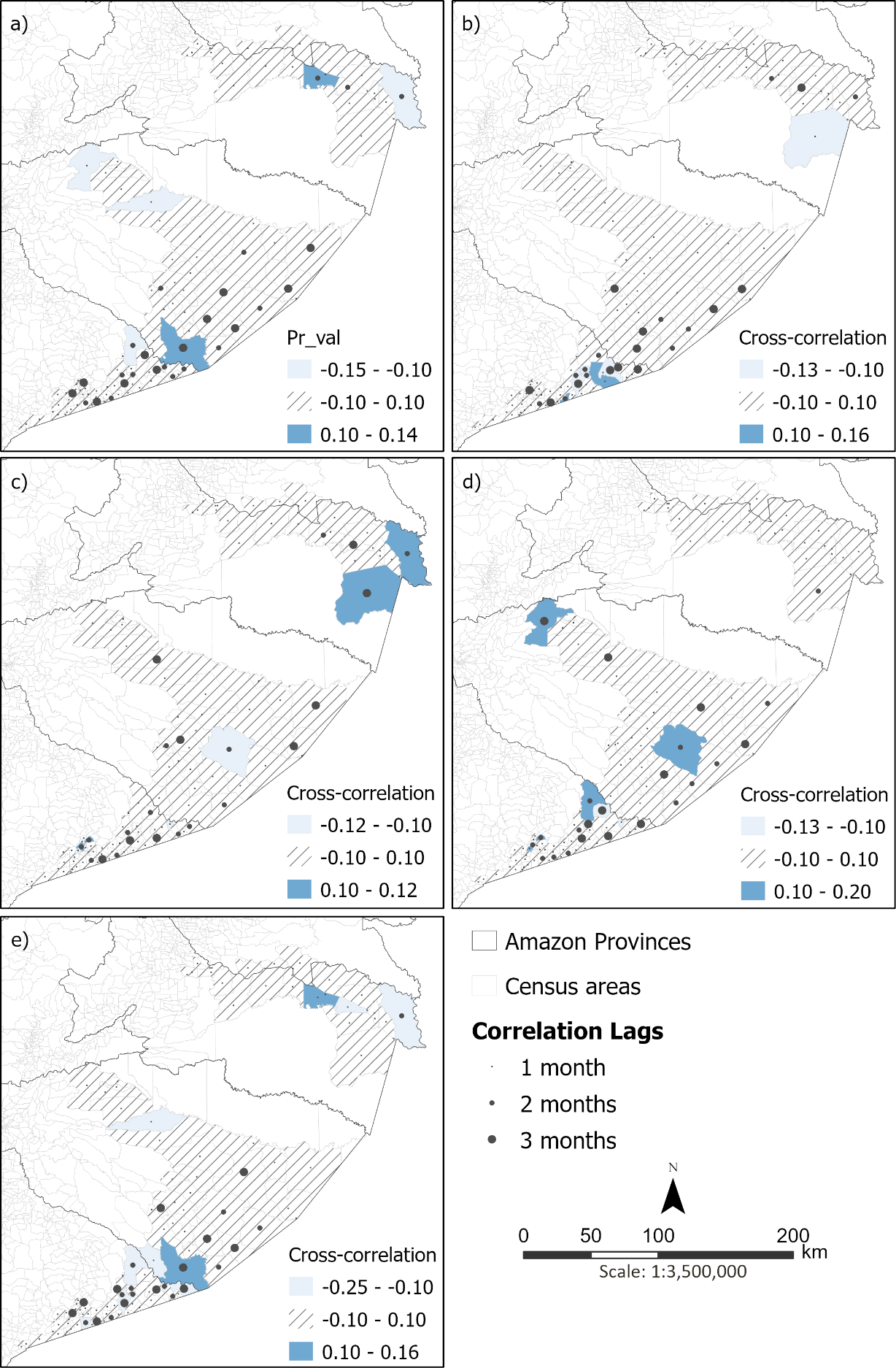
*

*Fig B. Cross-correlation values between Plasmodium falciparum incidence and a) Precipitation, b) Temperature, c) Soil Moisture, d) Terrestrial Water Content, and e) Runoff.* **Provinces and census areas borders were obtained from the public domain source** [**https://www.ecuadorencifras.gob.ec/documentos/web-inec/Geografia_Estadistica/Micrositio_geoportal/index.html**](https://www.ecuadorencifras.gob.ec/documentos/web-inec/Geografia_Estadistica/Micrositio_geoportal/index.html)**. Terms of use are described here** [**https://www.ecuadorencifras.gob.ec/documentos/web-inec/Geografia_Estadistica/Micrositio_geoportal/marco-geoestadistico-2022.html**](https://www.ecuadorencifras.gob.ec/documentos/web-inec/Geografia_Estadistica/Micrositio_geoportal/marco-geoestadistico-2022.html)
