# Supplementary material for: Impact of climate and land use/land cover changes on malaria incidence in the Ecuadorian Amazon
Source: PLOS Clim. Author manuscript; Available in PMC 2024 Jul 18. (PMC11257155; doi:10.1371/journal.pclm.0000315)
Supplement: Hypothesis about the relationship between hydro-climatic and LULC variables. — S1 Table. Hypothesis about the relationship between hydro-climatic and LULC variables. [file NIHMS2004284-supplement-Hypothesis_about_the_relationship_between_hydro-climatic_and_LULC_variables_.docx]

| **Factor** | **Indicator** | **Hypothesis** | **References** |
| --- | --- | --- | --- |
| **Climate** | Temperature | - A slight increase in temperature gives rise to a higher exponential transmission intensity, as it shortens the mosquito gonotropic cycle and reduces the larvarian development time. - Higher temperature acelerates the parasite development inside the vector and the sporocytes formation and benefits mosquitoes’ survival. | (1–4) |
|  | Precipitation | The increase in rainfall could negatively affect the abundance of mosquitos by flooding the breeding sites. Nevertheless, it could also provide the perfect aquatic medium for breeding sites.  The annual precipitation variability alters the vectors density. | (5–8) |
|  | Soil Moisture | In the dry season, larvae are known to occupy stagnant rivers, marshes, estuaries, irrigation canals, and rainwater pools. | (1,2,9,10) |
|  | Terrestrial Water content | Drives mosquitoes’ density as it is a key determinant for water availability | (11) |
|  | Surface Ruonoff | Reaches small-scale topographic depressions, which are breeding places for Anopheles mosquitoes |  |
| **Connectivity** | Road length | A higher number of roads could increase the connectivity, thus the contact frequency between humans coming from places with higher malaria incidences. | (12–14) |
|  | Distance to water bodies | Short distances could increase the contact of people with stagnant water bodies, which are optimal breeding sites for malaria mosquitos |  |
|  | Distance to forest frindges | Short distances could increase people's contact with places with higher entomological inoculation (e.g. infective bites) rates as fringes are optimal mosquito breeding sites. | (15–17) |
| **LULCC** | Deforestation | Alters local microclimate, especially temperature, through the change of albedo, evapotranspiration improving the habitat of mosquitos and their contact with humans. | (15,18–24) |
|  | Forest | Regulates temperature as it provides shade, limiting the development of mosquitos. | (6,25,26) |
|  | Agriculture | - At higher grassland, higher number of infectious bites. - Higher incidence of malaria in less crop diversification | (18,23,25) |
|  | Shrub vegetation | After deforestation, these are altered landscapes that present a higher abundance of mosquitos | (25) |
|  | Water Bodies | Mosquitos could reproduce in the edges of water bodies and feed from algae. | (19,23) |
|  | Urban Areas | Hot spots are created inside urban areas. These could contribute in the development of mosquitos and parasites. | (23) |

S1. Literature review

**References**

1. Pizzitutti F, Pan W, Barbieri A, Miranda JJ, Feingold B, Guedes GR, et al. A validated agent-based model to study the spatial and temporal heterogeneities of malaria incidence in the rainforest environment. Malar J. 2015;14(1):1–19.

2. Gunderson AK, Kumar RE, Recalde-Coronel C, Vasco LE, Valle-Campos A, Mena CF, et al. Malaria Transmission and Spillover across the Peru–Ecuador Border: A Spatiotemporal Analysis. Int J Environ Res Public Health. 2020;17(20):7434.

3. Lindsay SW, Birley MH. Climate change and malaria transmission. Ann Trop Med Parasitol. 1996;90(5):573–88.

4. Paaijmans KP, Blanford S, Bell AS, Blanford JI, Read AF, Thomas MB. Influence of climate on malaria transmission depends on daily temperature variation. Proceedings of the National Academy of Sciences. 2010;107(34):15135–9.

5. Breeland SG. Studies on the diurnal resting habits of Anopheles albimanus and A. pseudopunctipennis in El Salvador. Mosq News. 1972;32(1):99–106.

6. Pinault LL, Hunter FF. Malaria knowledge, concern, land management, and protection practices among land owners and/or managers in lowland versus highland Ecuador. Malar Res Treat. 2011;2011:1–13.

7. Rejmankova E, Roberts DR, Pawley A, Manguin S, Polanco J. Predictions of adult Anopheles albimanus densities in villages based on distances to remotely sensed larval habitats. Am J Trop Med Hyg. 1995;53(5):482–8.

8. Rejmankova E, Savage HM, Rejmanek M, Arredondo-Jimenez JI, Roberts DR. Multivariate analysis of relationships between habitats, environmental factors and occurrence of anopheline mosquito larvae Anopheles albimanus and A. pseudopunctipennis in southern Chiapas, Mexico. Journal of Applied Ecology. 1991;827–41.

9. Olson SH, Gangnon R, Elguero E, Durieux L, Guégan JF, Foley JA, et al. Links between climate, malaria, and wetlands in the Amazon Basin. Emerg Infect Dis. 2009;15(4):659.

10. Wolfarth-Couto B, Silva RA da, Filizola N. Variability in malaria cases and the association with rainfall and rivers water levels in Amazonas State, Brazil. Cad Saude Publica. 2019;35.

11. Girotto M, Rodell M. Terrestrial water storage. In: Extreme Hydroclimatic Events and Multivariate Hazards in a Changing Environment. Elsevier; 2019. p. 41–64.

12. Patz JA, Graczyk TK, Geller N, Vittor AY. Effects of environmental change on emerging parasitic diseases. Int J Parasitol. 2000;30(12–13):1395–405.

13. Koenraadt CJM, Paaijmans KP, Schneider P, Githeko AK, Takken W. Low larval vector survival explains unstable malaria in the western Kenya highlands. Tropical Medicine & International Health. 2006;11(8):1195–205.

14. Patz JA. Predicting key malaria transmission factors, biting and entomological inoculation rates, using modelled soil moisture in Kenya. Tropical Medicine & International Health. 1998;3(10):818–27.

15. MacDonald AJ, Mordecai EA. Amazon deforestation drives malaria transmission, and malaria burden reduces forest clearing. Proceedings of the National Academy of Sciences. 2019;116(44):22212–8.

16. De Castro MC, Monte-Mor RL, Sawyer DO, Singer BH. Malaria risk on the Amazon frontier. Proceedings of the National Academy of Sciences. 2006;103(7):2452–7.

17. Baeza A, Santos-Vega M, Dobson AP, Pascual M. The rise and fall of malaria under land-use change in frontier regions. Nat Ecol Evol. 2017;1(5):0108.

18. Vittor AY, Gilman RH, Tielsch J, Glass G, Shields TIM, Lozano WS, et al. The effect of deforestation on the human-biting rate of Anopheles darlingi, the primary vector of falciparum malaria in the Peruvian Amazon. Am J Trop Med Hyg. 2006;74(1):3–11.

19. Vittor AY, Pan W, Gilman RH, Tielsch J, Glass G, Shields T, et al. Linking deforestation to malaria in the Amazon: characterization of the breeding habitat of the principal malaria vector, Anopheles darlingi. Am J Trop Med Hyg [Internet]. 2009 Jul;81(1):5–12. Available from: https://pubmed.ncbi.nlm.nih.gov/19556558

20. Chaves LSM, Conn JE, López RVM, Sallum MAM. Abundance of impacted forest patches less than 5 km 2 is a key driver of the incidence of malaria in Amazonian Brazil. Sci Rep. 2018;8(1):1–11.

21. Barros FSM d, Arruda ME, Gurgel HC, Honório NA. Spatial clustering and longitudinal variation of Anopheles darlingi (Diptera: Culicidae) larvae in a river of the Amazon: the importance of the forest fringe and of obstructions to flow in frontier malaria. Bull Entomol Res. 2011;101(6):643–58.

22. Hahn MB, Gangnon RE, Barcellos C, Asner GP, Patz JA. Influence of deforestation, logging, and fire on malaria in the Brazilian Amazon. PLoS One. 2014;9(1):e85725.

23. Stefani A, Dusfour I, Corrêa APSA, Cruz MCB, Dessay N, Galardo AKR, et al. Land cover, land use and malaria in the Amazon: a systematic literature review of studies using remotely sensed data. Malar J. 2013;12(192):1–8.

24. Norris DE. Mosquito-borne diseases as a consequence of land use change. Ecohealth. 2004;1(1):19–24.

25. Aguilar Velasco HM, Salas B, Falconó C, Pazmiño X, Soria C. Malaria en la Amazonía ecuatoriana: estudio comparativo de la transmisión entre indígenas y colonos en el contexto de la organización espacial. Rev Inst Juan Cesar Garcia. 1993;22–38.

26. Olson SH, Gangnon R, Silveira GA, Patz JA. Deforestation and malaria in Mancio Lima county, Brazil. Emerg Infect Dis. 2010;16(7):1108.
